# Supplementary material for: Genetic determinants of gut microbiota composition and bile acid profiles in mice
Source: PLoS Genet. 2019 Aug 29;15(8):e1008073. doi: 10.1371/journal.pgen.1008073 (PMC6715156; doi:10.1371/journal.pgen.1008073)
Supplement: S1 Data — (PDF) [file pgen.1008073.s001.pdf]

Supporting Information

Supporting Data

Correlations among microbial, metabolite, and body weight traits

We found significant positive and negative associations between body weight and different Lachnospiraceae ESVs (S3 Table). This dual correlation is consistent with previous studies that have found this bacterial family to be positively (1) and negatively (2,3) correlated with obesity and other metabolic traits. Additionally, we identified significant associations between BAs and body weight. Body weight over time was inversely correlated with plasma levels of deoxycholic acid (DCA), taurochenodeoxycholic acid (TCDCA) and taurocholic acid (TCA). Conversely, cecal levels of muricholic acid (MCA) and ursodeoxycholic acid (UDCA) were positively correlated with body weight. The unconjugated plasma BAs allo-cholic acid (ACA), UDCA, 7-dehydrocholic acid (7-dHCA), hyodeoxycholic acid (HDCA), DCA, MCA and cholic acid (CA) were all positively associated with *Turicibacter* abundance. Interestingly, the only cecal bile acid to negatively correlate with *Turicibacter* was TCA ($r = -0.2619$, $p = 0.0035$). We also found several taxa among the Lachnospiraceae family were positively associated with conjugated secondary cecal bile acids including tauroursodeoxycholic acid (TUDCA), TCA, taurodeoxycholate (TDCA), glycodeoxycholic acid (GDCA), taurolithocholic acid (TLCA) and TCDCA. This is consistent with a previous study that found members of the Lachnospiraceae family were positively correlated with all secondary bile acids (4).

Corroboration of previous human and mouse genetic studies

Although recent studies show environment contributes more to the variability among gut microbiota composition than genetics (5–7), there are consistencies among different host organisms and geographically discrete populations indicate specific taxa and related traits are

under the influence of the host genome. Our results in the DO population corroborate several of these key findings for both microbial and clinical traits. These shared findings can be followed up for mechanistic experiments.

We observed the strongest associations to the host genome with members of the Firmicutes phyla, including unknown members of the Clostridiales order, the Lachnospiraceae, Christensenellacae and S24-7 families, the *Turicibacter* and *Coprococcus* genera, as well as the species *Akkermansia muciniphila* and *Ruminococcus gnavus*. These taxa have consistently been identified in multiple studies as either highly heritable or associating to positions on the host genome (1,8–12). Furthermore, our study replicated correlations between taxa including the Peptostreptococcaeae and Turicibacteraceae families (13), which may give insight into microbial dynamics that govern bile acid profiles.

Although the majority of these shared taxa seen in our study did not map to the same loci as in previous studies, we did find several microbial taxa and clinical traits that mapped to the same position of the mouse genome as in previous studies. We did find several clinical QTL in the DO population that co-mapped with clinical QTL previously identified in the HMDP population. For example, we found a QTL for body weight at 14 weeks on chr 2 at 135.2 that overlaps with a percent body fat increase QTL between 138.9 - 139.4 Mbp (14). We also found a QTL for fat pad weight on chr 7 at ~40 Mbp that falls within the same confidence interval as a HMDP QTL for triglyceride (TG) gonadal fat (1). Additionally, QTL for taxa classified to the Coriobacteraceae family mapped to chr 10 between ~116 – 120 Mbp in our study and in an advanced intercross line used by Benson et al. (8) (S1 Table). However, the majority of these shared taxa seen in our study and previous analysis did not map to the same position.

Furthermore, our study replicated correlations between taxa including the Peptostreptococcaeae and Turicibacteriaceae families (13). Previous studies in humans and rats

also identified a significant correlation between these taxa (13,15), and both taxa are consistently identified as heritable in humans and mice (2,8,13). This correlation is particularly notable since we found that these two organisms have complimentary BA metabolism capabilities, where the Turicibacteriaceae family performs the deconjugation necessary for members of Peptostreptococcaceae to epimerize bile acids. BAs must be deconjugated prior to epimerization, so Peptostreptococcaeae may associate with Turicibacteriaceae in order to utilize this metabolic capability. Thus, their co-occurrence may provide a fitness advantage for small intestine colonization. These findings may give insight into microbial dynamics that govern BA profiles and warrant further investigation.

Given the high degree of variability in the gut microbiome across subjects and host organisms, these instances of congruence between studies argues that there are specific taxa responsive to host genotype that may warrant follow-up investigation. Our work with the DO population provides an approach to validate these associations.

Overlapping bacterial and bile acid QTL

We found QTL for an unknown genus in the Peptostreptococcaceae family overlapping with the hotspot containing QTL for plasma levels of CA, CDCA, UDCA, MCA, 7-dHCA and glycodehydrocholic acid (G-dHCA) on chr 3 between ~40-50 Mbp. These QTL all show the same founder strain haplotype effects, where the NOD haplotype is associated with higher levels of these traits (S6A-S6F Fig). *Peptostreptococcus productus*, a member of the Peptostreptococcaceae family, has 3α-, 3β-, and 7β-hydroxysteroid dehydrogenases and is capable of oxidation and epimerization of BAs (16). Several of these secondary bile acids require 7β-epimerization, including hyocholic acid (HCA) and UDCA which is produced from 7β-epimerization of CDCA (17), which may help explain why these BAs co-map with Peptostreptococcaceae abundance. An

interesting candidate within the QTL peak region is progesterone receptor membrane component 2 (*Pgrmc2*), which is expressed in bile sensitive tissues such as intestine, liver and brown adipose (18). PGRMC2 is predicted to be a membrane receptor (19), which binds to P450 cytochrome proteins and has similar characteristics to PGRMC1 (20). The shared sequence between *Pgrmc2* and *Pgrmc1* is especially interesting in the context of BAs because *Pgrmc1* directly binds to Cyp7a1 (21), a P450 cytochrome protein responsible for the regulation of BA synthesis. These data suggest that *Pgrmc2* may be a novel gene involved in BA signaling and/or homeostasis.

On chr 1 at ~90 – 100 Mbp, we identified overlapping QTL for *Akkermansia muciniphila* and plasma levels of CA, MCA and 7-dHCA, where the NZO haplotype is positively associated, and the 129 haplotype is negatively associated with each of these traits (S7A-S7D Fig). Significant positive correlations were also found between the abundance of *A. muciniphila* and plasma levels of CA ($r = 0.19$, $p < 0.0045$) and MCA ($r = 0.17$, $p < 0.0149$) (S7F-S7G Fig). These observations are particularly striking given the recent studies associating the abundance of *A. muciniphila* and BAs. For example, Pierre et al. found the abundance of *A. muciniphila* was positively correlated with higher levels of circulating primary bile acids (22) and administration of the secondary bile acid UDCA was found to increase its abundance (23). Furthermore, supplementation with up to 1% porcine bile extract increased *A. muciniphila* growth *in vivo* (24). In the intestine, *A. muciniphila* degrades host mucins (25), which provide growth substrates for other intestinal commensals (26). Notably, BAs have a stimulatory effect on mucin secretion as a defense mechanism to protect the gastrointestinal epithelium against potential BA toxicity (27,28). Therefore, the positive correlation between bile acid levels and *A. muciniphila* may be the result of this stimulatory effect where greater mucin secretion from BA stimulation can support a larger intestinal *A. muciniphila* population.

There is growing interest in the potential therapeutic role of *A. muciniphila* since it has been associated with improvements in host metabolic syndrome (1,29–31) and plays a key role in regulating intestinal barrier function and mucosal immunity (29,32). Strikingly, we found several candidate genes under the QTL region on chr 1 related to host lipid metabolism and immunity (S7E Fig). Top immune-related genes include *Lrrfip1*, a transcription regulator of TLR pathway signaling (33) and TNF expression (34), and *Gpr35*, a G protein-coupled receptor for the mucosal chemokine CXCL17 (35). Candidate lipid metabolism genes include *Farp2* and *Stk25*, which were previously identified as candidate genes for plasma HDL levels (36). In fact, several mouse studies using F2 crosses have identified QTL for plasma cholesterol and HDL levels at this position on chr 1 (36–39) including one where the association was driven by the 129 haplotype (38). The plasma HDL QTL found at the position as the microbial and metabolite QTL on chr 1 is particularly interestingly because *A. muciniphila* abundance has been associated with elevated HDL levels (40) and administration of a purified protein from this microbe decreased HDL and LDL cholesterol levels, indicating it may have a regulatory impact on cholesterol metabolism (30). Therefore, the co-mapping *A. muciniphila* and plasma bile acid traits seen in our study may be driven by another unmeasured factor or plasma lipid, which explains why they map to the same position. Future integration of additional lipid profiling may identify a causal factor that explains the relationship between these microbial and bile acid traits.

Supporting Data References

1. Org E, Parks BW, Joo JWJ, Emert B, Schwartzman W, Kang EY, et al. Genetic and environmental control of host-gut microbiota interactions. Genome Res. 2015 Oct 1;25(10):1558–69.
2. O'Connor A, Quizon PM, Albright JE, Lin FT, Bennett BJ. Responsiveness of

cardiometabolic-related microbiota to diet is influenced by host genetics. Mamm Genome. 2014 Dec 1;25(11–12):583–99.

3. Kreznar JH, Keller MP, Traeger LL, Rabaglia ME, Schueler KL, Stapleton DS, et al. Host Genotype and Gut Microbiome Modulate Insulin Secretion and Diet-Induced Metabolic Phenotypes. Cell Rep. 2017 Feb 14;18(7):1739–50.
4. Theriot CM, Bowman AA, Young VB. Antibiotic-Induced Alterations of the Gut Microbiota Alter Secondary Bile Acid Production and Allow for Clostridium difficile Spore Germination and Outgrowth in the Large Intestine. Ellermeier CD, editor. mSphere. 2016 Jan 1;1(1):e00045-15.
5. Rothschild D, Weissbrod O, Barkan E, Kurilshikov A, Korem T, Zeevi D, et al. Environment dominates over host genetics in shaping human gut microbiota. Nature. 2018 Mar 8;555(7695):210–5.
6. Zhernakova A, Kurilshikov A, Bonder MJ, Tigchelaar EF, Schirmer M, Vatanen T, et al. Population-based metagenomics analysis reveals markers for gut microbiome composition and diversity. Science. 2016 Apr 29;352(6285):565–9.
7. Falony G, Joossens M, Vieira-Silva S, Wang J, Darzi Y, Faust K, et al. Population-level analysis of gut microbiome variation. Science. 2016 Apr 29;352(6285):560–4.
8. Benson AK, Kelly SA, Legge R, Ma F, Low SJ, Kim J, et al. Individuality in gut microbiota composition is a complex polygenic trait shaped by multiple environmental and host genetic factors. Proc Natl Acad Sci U S A. 2010 Nov 2;107(44):18933–8.
9. Wang J, Thingholm LB, Skiecevičienė J, Rausch P, Kummen M, Hov JR, et al. Genome-wide association analysis identifies variation in vitamin D receptor and other host factors influencing the gut microbiota. Nat Genet. 2016 Nov 1;48(11):1396–406.
10. Davenport ER, Cusanovich DA, Michelini K, Barreiro LB, Ober C, Gilad Y. Genome-

Wide Association Studies of the Human Gut Microbiota. White BA, editor. PLoS One. 2015 Jan 1;10(11):e0140301.

11. Leamy LJ, Kelly SA, Nietfeldt J, Legge RM, Ma F, Hua K, et al. Host genetics and diet, but not immunoglobulin A expression, converge to shape compositional features of the gut microbiome in an advanced intercross population of mice. Genome Biol. 2014 Jan 1;15(12):552.

12. McKnite AM, Perez-Munoz ME, Lu L, Williams EG, Brewer S, Andreux PA, et al. Murine gut microbiota is defined by host genetics and modulates variation of metabolic traits. White BA, editor. PLoS One. 2012 Jan 1;7(6):e39191.

13. Goodrich JK, Davenport ER, Beaumont M, Jackson MA, Knight R, Ober C, et al. Genetic Determinants of the Gut Microbiome in UK Twins. Cell Host Microbe. 2016 May 11;19(5):731–43.

14. Parks BW, Nam E, Org E, Kostem E, Norheim F, Hui ST, et al. Genetic control of obesity and gut microbiota composition in response to high-fat, high-sucrose diet in mice. Cell Metab. 2013 Jan 8;17(1):141–52.

15. Li D, Chen H, Mao B, Yang Q, Zhao J, Gu Z, et al. Microbial Biogeography and Core Microbiota of the Rat Digestive Tract. Sci Rep. 2017 Apr 4;8(1):45840.

16. Edenharder R, Pfützner A, Hammann R. Characterization of NAD-dependent 3 alpha- and 3 beta-hydroxysteroid dehydrogenase and of NADP-dependent 7 beta-hydroxysteroid dehydrogenase from Peptostreptococcus productus. Biochim Biophys Acta. 1989 Aug 8;1004(2):230–8.

17. Wahlström A, Sayin SI, Marschall H-U, Bäckhed F. Intestinal Crosstalk between Bile Acids and Microbiota and Its Impact on Host Metabolism. Cell Metab. 2016 Jun 15;24(1):41–50.

18. Chen C, Sargent C, Quilter C, Yang Z, Ren J, Affara N, et al. Cloning, mapping and molecular characterization of porcine progesterone receptor membrane component 2 (PGRMC2) gene. Genet Mol Biol. 2010 Jul 1;33(3):471–4.

19. Gerdes D, Wehling M, Leube B, Falkenstein E. Cloning and tissue expression of two putative steroid membrane receptors. Biol Chem. 1998 Jul 1;379(7):907–11.

20. Wendler A, Wehling M. PGRMC2, a yet uncharacterized protein with potential as tumor suppressor, migration inhibitor, and regulator of cytochrome P450 enzyme activity. Steroids. 2013 Jun 1;78(6):555–8.

21. Hughes AL, Powell DW, Bard M, Eckstein J, Barbuch R, Link AJ, et al. Dap1/PGRMC1 binds and regulates cytochrome P450 enzymes. Cell Metab. 2007 Feb 1;5(2):143–9.

22. Pierre JF, Martinez KB, Ye H, Nadimpalli A, Morton TC, Yang J, et al. Activation of bile acid signaling improves metabolic phenotypes in high-fat diet-induced obese mice. Am J Physiol Gastrointest Liver Physiol. 2016 Aug 1;311(2):G286-304.

23. Van den Bossche L, Hindryckx P, Devisscher L, Devriese S, Van Welden S, Holvoet T, et al. Ursodeoxycholic Acid and Its Taurine- or Glycine-Conjugated Species Reduce Colitogenic Dysbiosis and Equally Suppress Experimental Colitis in Mice. Elkins CA, editor. Appl Environ Microbiol. 2017 Apr 1;83(7):e02766-16.

24. van der Ark KCH, Nugroho ADW, Berton-Carabin C, Wang C, Belzer C, de Vos WM, et al. Encapsulation of the therapeutic microbe Akkermansia muciniphila in a double emulsion enhances survival in simulated gastric conditions. Food Res Int. 2017 Dec 1;102:372–9.

25. Derrien M, Vaughan EE, Plugge CM, de Vos WM. Akkermansia muciniphila gen. nov., sp. nov., a human intestinal mucin-degrading bacterium. Int J Syst Evol Microbiol. 2004 Sep 1;54(Pt 5):1469–76.

26. Belzer C, de Vos WM. Microbes inside--from diversity to function: the case of Akkermansia. ISME J. 2012 Aug 1;6(8):1449–58.

27. Shekels LL, Lyftogt CT, Ho SB. Bile acid-induced alterations of mucin production in differentiated human colon cancer cell lines. Int J Biochem Cell Biol. 1996 Feb 1;28(2):193–201.

28. Klinkspoor JH, Mok KS, Van Klinken BJW, Tytgat GNJ, Lee SP, Groen AK. Mucin secretion by the human colon cell line LS174T is regulated by bile salts. Glycobiology. 1999 Jan 1;9(1):13–9.

29. Everard A, Belzer C, Geurts L, Ouwerkerk JP, Druart C, Bindels LB, et al. Cross-talk between Akkermansia muciniphila and intestinal epithelium controls diet-induced obesity. Proc Natl Acad Sci U S A. 2013 May 28;110(22):9066–71.

30. Plovier H, Everard A, Druart C, Depommier C, Van Hul M, Geurts L, et al. A purified membrane protein from Akkermansia muciniphila or the pasteurized bacterium improves metabolism in obese and diabetic mice. Nat Med. 2017 Jan 1;23(1):107–13.

31. Cani PD, de Vos WM. Next-Generation Beneficial Microbes: The Case of Akkermansia muciniphila. Front Microbiol. 2017 Jan 1;8:1765.

32. Derrien M, Van Baarlen P, Hooiveld G, Norin E, Müller M, de Vos WM. Modulation of Mucosal Immune Response, Tolerance, and Proliferation in Mice Colonized by the Mucin-Degrader Akkermansia muciniphila. Front Microbiol. 2011 Jan 1;2:166.

33. Arakawa R, Bagashev A, Song L, Maurer K, Sullivan KE. Characterization of LRRFIP1. Biochem Cell Biol. 2010 Dec 1;88(6):899–906.

34. Shi L, Song L, Fitzgerald M, Maurer K, Bagashev A, Sullivan KE. Noncoding RNAs and LRRFIP1 regulate TNF expression. J Immunol. 2014 Apr 1;192(7):3057–67.

35. Maravillas-Montero JL, Burkhardt AM, Hevezi PA, Carnevale CD, Smit MJ, Zlotnik A.

Cutting edge: GPR35/CXCR8 is the receptor of the mucosal chemokine CXCL17. J Immunol. 2015 Jan 1;194(1):29–33.

36. Su Z, Cox A, Shen Y, Stylianou IM, Paigen B. Farp2 and Stk25 are candidate genes for the HDL cholesterol locus on mouse chromosome 1. Arterioscler Thromb Vasc Biol. 2009 Jan 1;29(1):107–13.

37. Purcell-Huynh DA, Weinreb A, Castellani LW, Mehrabian M, Doolittle MH, Lusis AJ. Genetic factors in lipoprotein metabolism. Analysis of a genetic cross between inbred mouse strains NZB/BINJ and SM/J using a complete linkage map approach. J Clin Invest. 1995 Oct 1;96(4):1845–58.

38. Ishimori N, Li R, Kelmenson PM, Korstanje R, Walsh KA, Churchill GA, et al. Quantitative trait loci analysis for plasma HDL-cholesterol concentrations and atherosclerosis susceptibility between inbred mouse strains C57BL/6J and 129S1/SvImJ. Arterioscler Thromb Vasc Biol. 2004 Jan 1;24(1):161–6.

39. Su Z, Ishimori N, Chen Y, Leiter EH, Churchill GA, Paigen B, et al. Four additional mouse crosses improve the lipid QTL landscape and identify Lipg as a QTL gene. J Lipid Res. 2009 Oct 1;50(10):2083–94.

40. Fu J, Bonder MJ, Cenit MCMC, Tigchelaar EF, Maatman A, Dekens JAMM, et al. The Gut Microbiome Contributes to a Substantial Proportion of the Variation in Blood Lipids. Circ Res. 2015 Oct 9;117(9):817–24.

Supporting Tables

S1 Table. Measures of variability of microbial exact sequence variants (ESVs) or taxon (phylum, class, order, family, genus) in DO mice. Data presented as normalized read counts; n = 399; SD, standard deviation.

S2 Table. Measures of variability of cecal and plasma bile acids in DO mice. Bile acid levels are presented as log2(peak area); n = 384; SD, standard deviation.

S3 Table. Correlations among microbial taxa, bile acid and weight traits. Spearman's rank correlation. Only microbial exact sequence variants, genera and family included in figure. Correlations shown passed FDR < 0.01 cut-off and correlation coefficient either < -0.35 or > 0.35. Correlating bile acids from same tissue removed from table for brevity.

S4 Table. QTL peaks for gut microbiota, plasma and cecal bile acid, and weight traits in the Diversity Outbred mice. Only QTL with LOD > 5.5 shown. "Pos" is peak position is Mbp. "ci_lo" and "ci_hi" correspond to the positions for the 95% bayesian confidence interval.

S5 Table. Media used for bacterial culture. Medium 14(b) recipe.

Supporting Figures

S1 Figure. Principal coordinate analysis (PCoA) of unweighted UniFrac distances for fecal samples. PCoA shows significant clustering by (A) sex (F = 5.572, p = 0.001) and (B) wave (F = 16.954, p = 0.001). Clustering by treatment evaluated by PERMANOVA.

S2 Figure. Plasma and cecal bile acids group by sex, but not wave. PCAs of plasma bile acid profiles colored by (A) sex ($p < 0.0001$) and (B) wave ($p = 0.594$), and PCAs of cecal bile acid profiles colored by (C) sex ($p = 0.011$) and (D) wave ($p = 0.207$). Kruskal Wallis one-way test followed by Wilcoxon pair-wise multiple comparisons with Benjamini and Hochberg correction.

S3 Figure. Related bile acid species map associate to same locus. (A) Haplotype effects and LOD scores of plasma taurodeoxycholic acid (TDCA), (B) cecal deoxycholic acid (DCA), (C) cecal isodeoxycholic acid (IDCA) and (D) cecal hyodeoxycholic acid (HDCA). For each plot, the x-axis is the physical position in Mbp along chr 12. The y-axis for the top panel is the effect coefficient depicting the estimated contributions of each founder allele, and the y-axis in the bottom panel is the LOD score. (E) Cecal levels of isolithocholic acid (ILCA) and lithocholic acid (LCA) associate to same locus on chr 11. (F) Estimated founder allele effects for cecal ILCA and (G) LCA. (H) Genes under cecal LCA and ILCA QTL interval. Dashed lines denote QTL confidence interval.

S4 Figure. Gut associated bacteria have differential growth responses to conjugated bile acids. Growth rate in the presence of 1 mM conjugated bile acids or methanol control for (A) *Bacteroides thetaiotaomicron*, (B) *Clostridium asparagiforme*, (C) *Escherichia coli* MS200-1, and (D) *Lactobacillus reuteri.* Data shown are from duplicate experiments with three technical replicates. Data are presented as mean ± SEM; Welch's *t* test; no significant differences were observed between growth conditions for any of the tested organisms.

S5 Figure. Peptostreptococcaceae and plasma bile acids co-map on chromosome (chr) 3. Haplotype effects and LOD scores of (A) Peptostreptococcaceae family, (B) plasma cholic acid

(CA), (C) plasma chenodeoxycholic acid (CDCA), (D) plasma muricholic acid (MCA), (E) plasma ursodeoxycholic acid (UDCA), and (F) plasma 7-dehydrocholic acid (7-dHCA). For each plot, the x-axis is the physical position in Mbp along chr 3. The y-axis for the top panel is the effect coefficient depicting the estimated contributions of each founder allele, and the y-axis in the bottom panel is the LOD score. All overlapping QTL have positive association with the NOD allele. (G) Protein coding genes under QTL interval.

S6 Figure. Exact sequence variant of *Akkermansia muciniphila* and plasma bile acid QTL overlap on chromosome (chr) 1. Haplotype effects and LOD scores of (A) *A. muciniphila* (B) plasma cholic acid (CA), (C) plasma muricholic acid (MCA), and (D) plasma 7-dehydrocholic acid (7-dHCA). For each plot, the x-axis is the physical position in Mbp along chr 1. The y-axis for the top panel is the effect coefficient depicting the estimated contributions of each founder allele, and the y-axis in the bottom panel is the LOD score. (E) Protein coding genes under 10 Mbp QTL interval. Spearman correlations in the DO mice between *A. muiniphila* and (F) plasma CA, (G) plasma MCA, and (H) plasma 7-dHCA levels. Correlation p-values adjusted for multiple tests using Benjamini and Hochberg correction. Higher levels of these microbial and bile acid traits were associated with the NZO haplotype and lower levels were associated with the 129 haplotype. (E) Protein coding genes under 10 Mbp QTL interval. Dashed lines denote QTL confidence interval. Spearman correlations in the DO mice between *A. muiniphila* and (F) plasma CA, (G) plasma MCA, and (H) plasma 7-dHCA levels. Correlation p-values adjusted for multiple tests using Benjamini and Hochberg correction.
