# Supplementary figures and images for: Genetic determinants of gut microbiota composition and bile acid profiles in mice
Source: PLoS Genet. 2019 Aug 29;15(8):e1008073. doi: 10.1371/journal.pgen.1008073 (PMC6715156; doi:10.1371/journal.pgen.1008073)

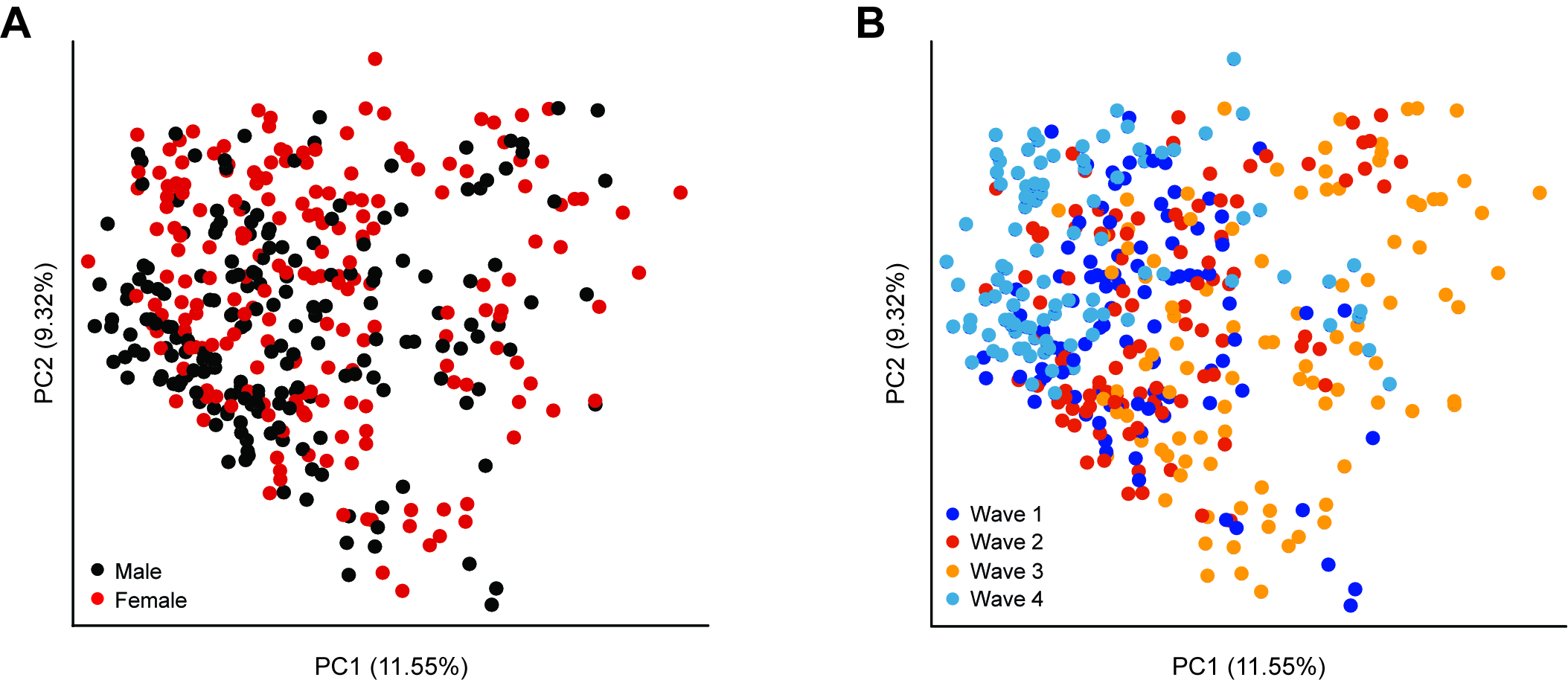

Supplement: S1 Fig — PCoA shows significant clustering by (A) sex (F = 5.572, p = 0.001) and (B) wave (F = 16.954, p = 0.001). Clustering by treatment evaluated by PERMANOVA. (TIF) [file pgen.1008073.s002.tif]

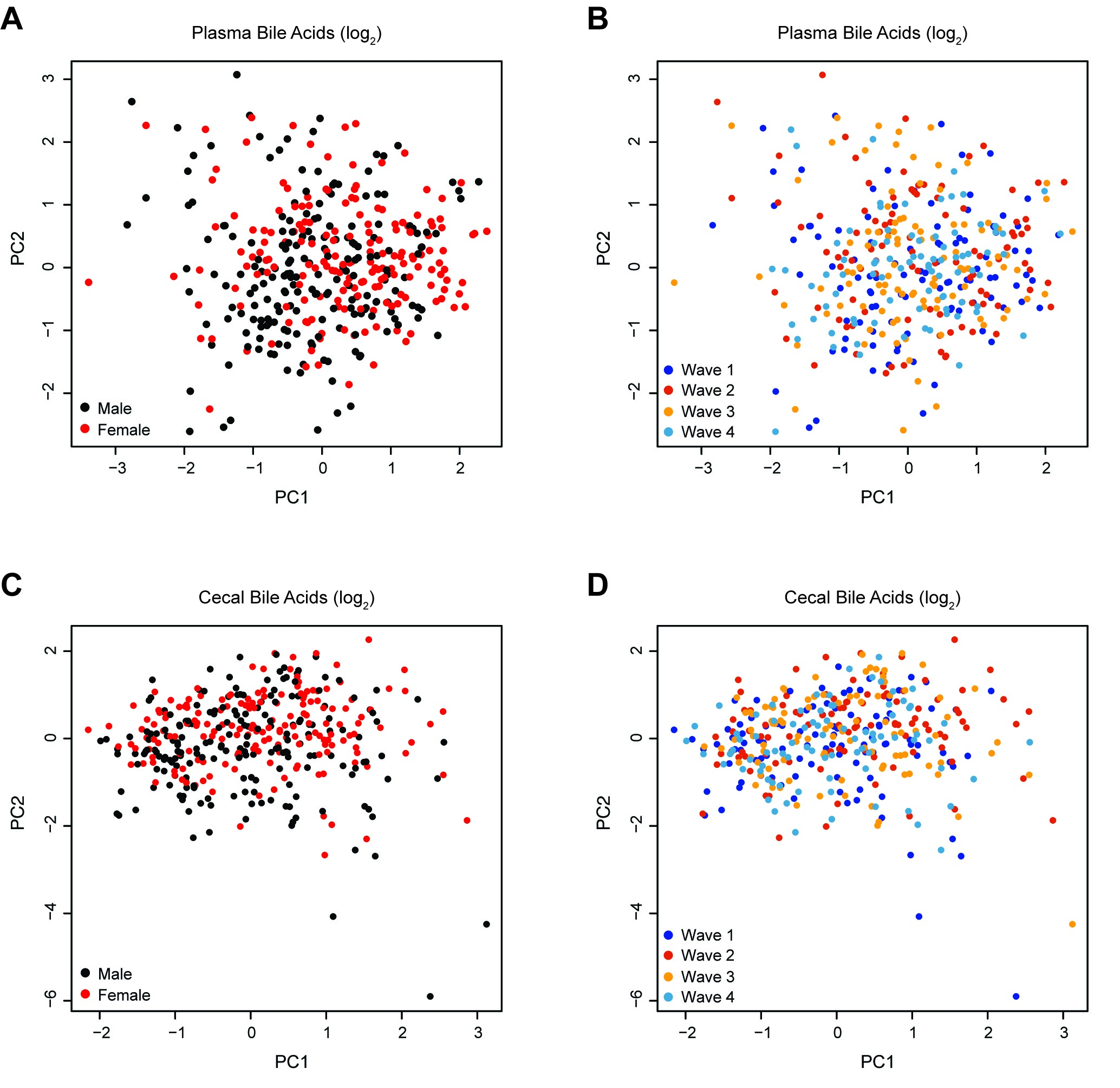

Supplement: S2 Fig — PCAs of plasma bile acid profiles colored by (A) sex (p < 0.0001) and (B) wave (p = 0.594), and PCAs of cecal bile acid profiles colored by (C) sex (p = 0.011) and (D) wave (p = 0.207). Kruskal Wallis one-way test followed by Wilcoxon pair-wise multiple comparisons with Benjamini and Hochberg correction. (TIF) [file pgen.1008073.s003.tif]

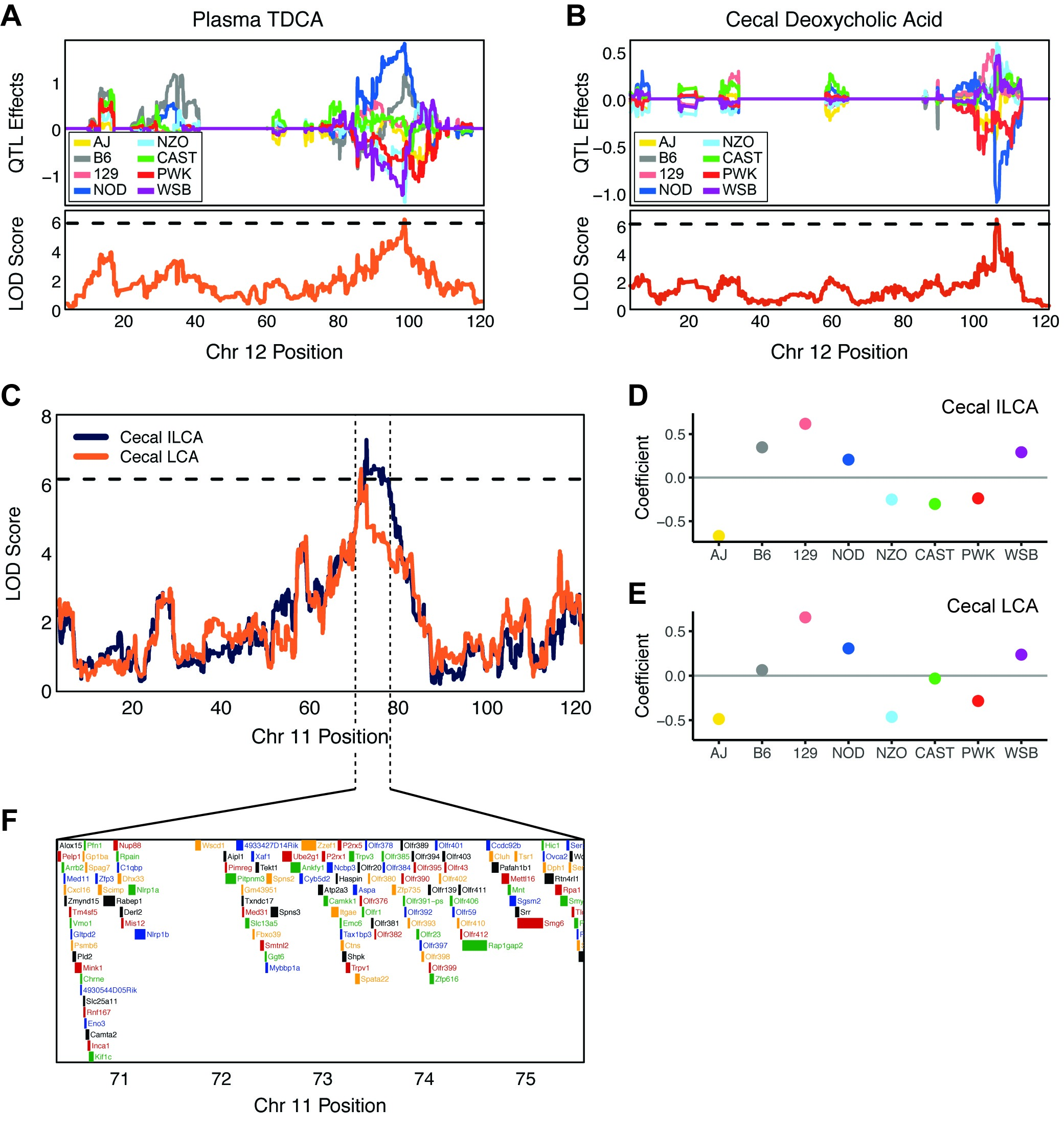

Supplement: S3 Fig — (A) Haplotype effects and LOD scores of plasma taurodeoxycholic acid (TDCA) and (B) cecal deoxycholic acid (DCA). For each plot, the x-axis is the physical position in Mbp along chr 12. The y-axis for the top panel is the effect coefficient depicting the estimated contributions of each founder allele, and the y-axis in the bottom panel is the LOD score. (C) Cecal levels of isolithocholic acid (ILCA) and lithocholic acid (LCA) associate to same locus on chr 11. (D) Estimated founder allele effects for cecal ILCA and (E) LCA. (F) Genes under cecal LCA and ILCA QTL interval. Vertical dashed lines denote QTL confidence interval. Horizontal dashed lines correspond to LOD = 6.11 (p < 0.5). (TIF) [file pgen.1008073.s004.tif]

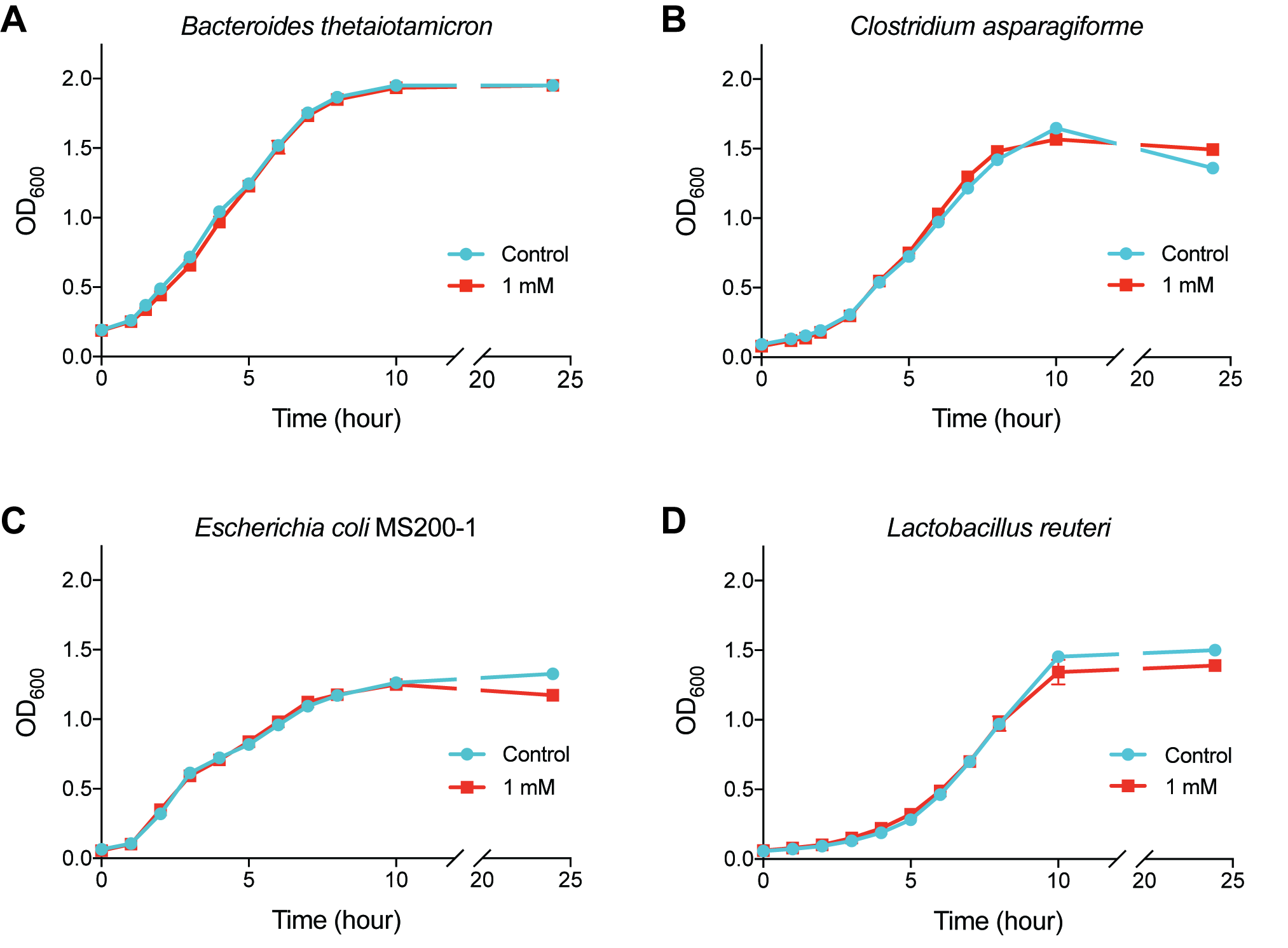

Supplement: S4 Fig — Growth rate in the presence of 1 mM conjugated bile acids or methanol control for (A) Bacteroides thetaiotaomicron, (B) Clostridium asparagiforme, (C) Escherichia coli MS200-1, and (D) Lactobacillus reuteri. Data shown are from duplicate experiments with three technical replicates. Data are presented as mean ± SEM; Welch’s t test; no significant differences were observed between growth conditions for any of the tested organisms. (TIF) [file pgen.1008073.s005.tif]

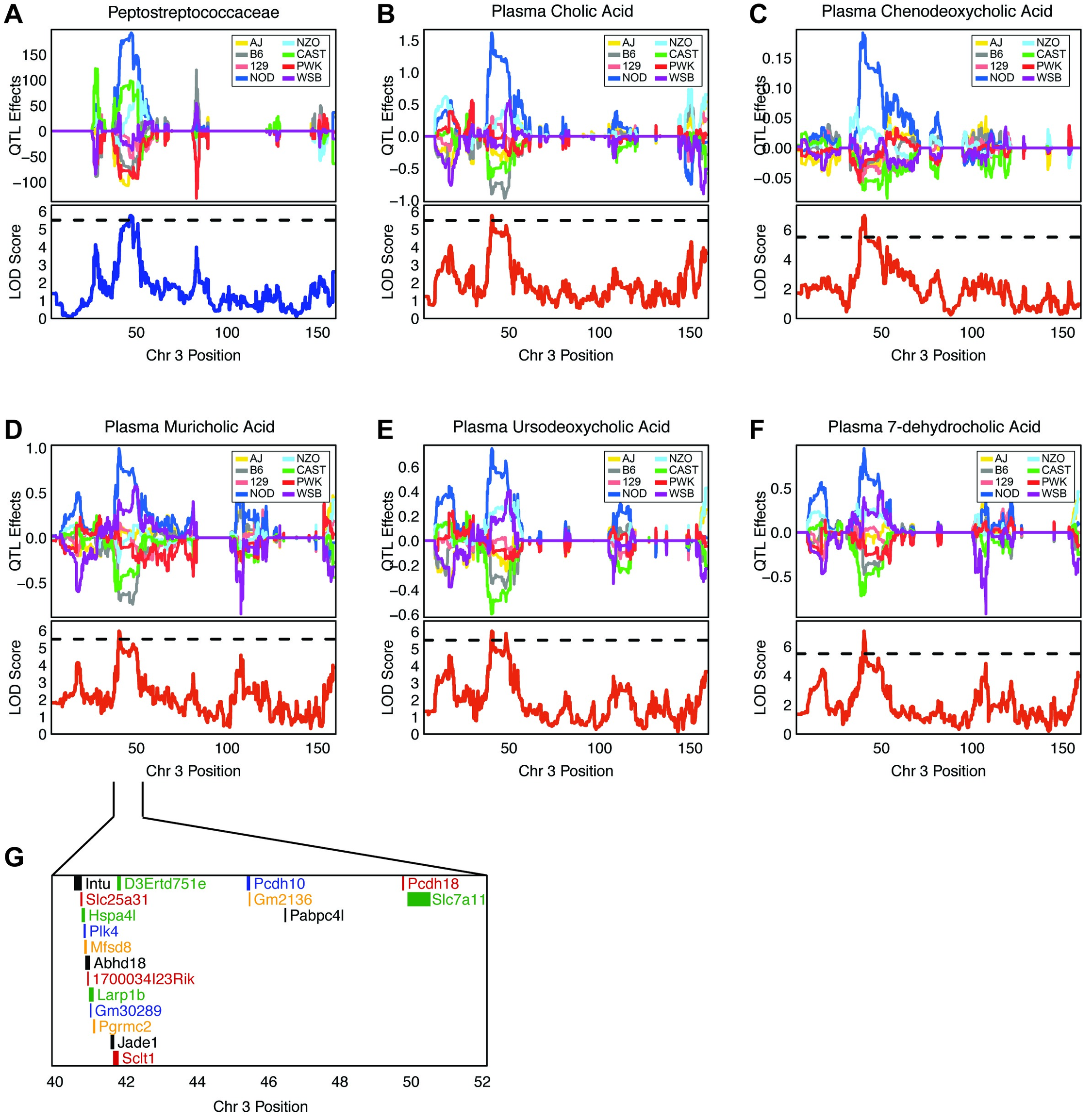

Supplement: S5 Fig — Haplotype effects and LOD scores of (A) Peptostreptococcaceae family, (B) plasma cholic acid (CA), (C) plasma chenodeoxycholic acid (CDCA), (D) plasma muricholic acid (MCA), (E) plasma ursodeoxycholic acid (UDCA), and (F) plasma 7-dehydrocholic acid (7-dHCA). For each plot, the x-axis is the physical position in Mbp along chr 3. The y-axis for the top panel is the effect coefficient depicting the estimated contributions of each founder allele, and the y-axis in the bottom panel is the LOD score. Horizontal dashed line corresponds to LOD = 5.5. All overlapping QTL have positive association with the NOD allele. (G) Protein coding genes under QTL interval. (TIF) [file pgen.1008073.s006.tif]

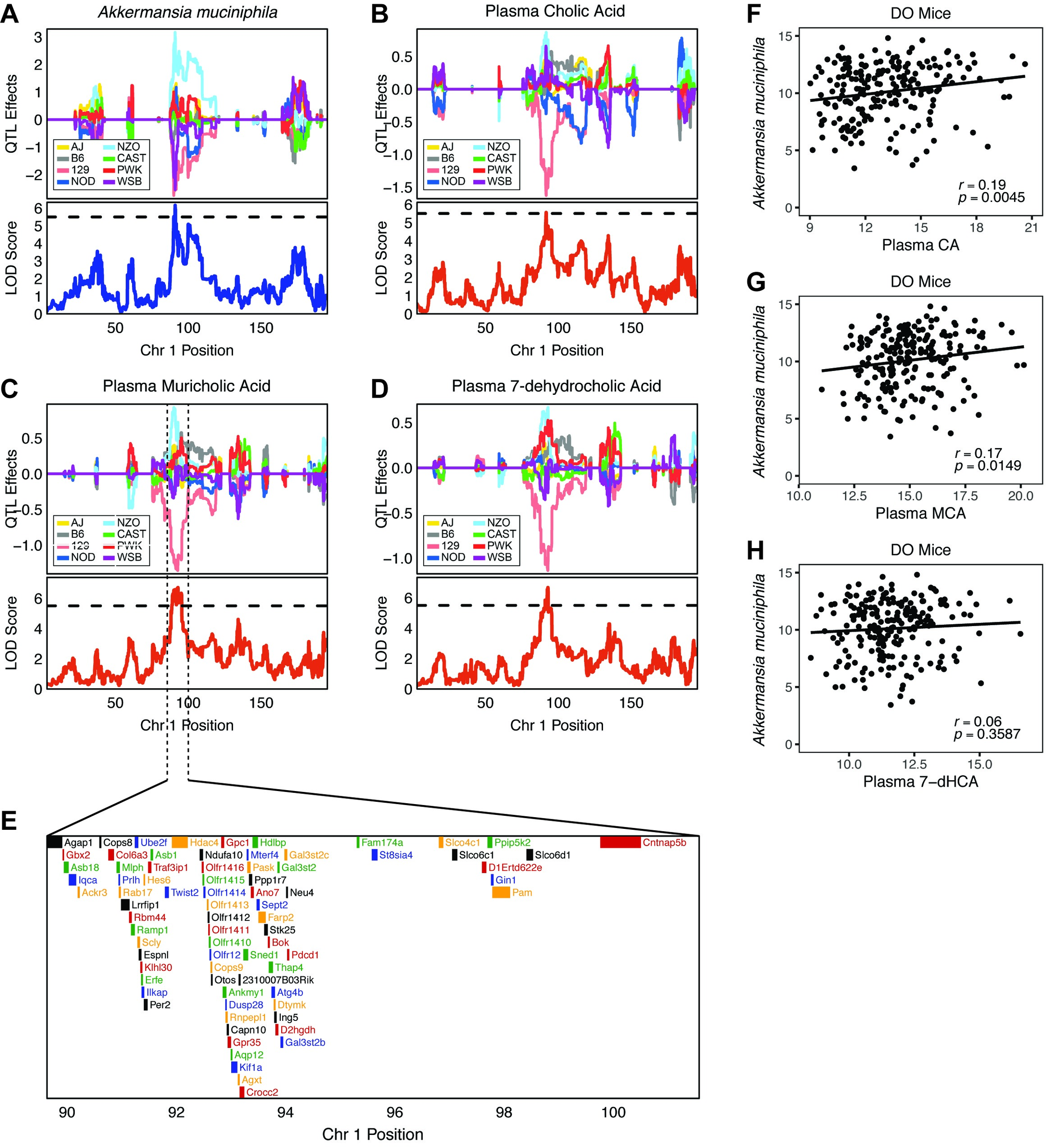

Supplement: S6 Fig — Haplotype effects and LOD scores of (A) A. muciniphila (B) plasma cholic acid (CA), (C) plasma muricholic acid (MCA), and (D) plasma 7-dehydrocholic acid (7-dHCA). For each plot, the x-axis is the physical position in Mbp along chr 1. The y-axis for the top panel is the effect coefficient depicting the estimated contributions of each founder allele, and the y-axis in the bottom panel is the LOD score. Horizontal dashed line corresponds to LOD = 5.5. (E) Protein coding genes under 10 Mbp QTL interval. Spearman correlations in the DO mice between A. muiniphila and (F) plasma CA, (G) plasma MCA, and (H) plasma 7-dHCA levels. Correlation p-values adjusted for multiple tests using Benjamini and Hochberg correction. Higher levels of these microbial and bile acid traits were associated with the NZO haplotype and lower levels were associated with the 129 haplotype. (E) Protein coding genes under 10 Mbp QTL interval. Dashed lines denote QTL confidence interval. Spearman correlations in the DO mice between A. muiniphila and (F) plasma CA, (G) plasma MCA, and (H) plasma 7-dHCA levels. Correlation p-values adjusted for multiple tests using Benjamini and Hochberg correction. (TIF) [file pgen.1008073.s007.tif]
